# The Distribution and Role of the CFTR Protein in the Intracellular Compartments

**DOI:** 10.3390/membranes11110804

**Published:** 2021-10-22

**Authors:** Agnieszka Lukasiak, Miroslaw Zajac

**Affiliations:** Department of Physics and Biophysics, Institute of Biology, Warsaw University of Life Sciences—SGGW, 02-776 Warsaw, Poland; miroslaw_zajac@sggw.edu.pl

**Keywords:** chloride channels, cystic fibrosis transmembrane conductance regulator, ion transport, intracellular organelle

## Abstract

Cystic fibrosis is a hereditary disease that mainly affects secretory organs in humans. It is caused by mutations in the gene encoding CFTR with the most common phenylalanine deletion at position 508. CFTR is an anion channel mainly conducting Cl^−^ across the apical membranes of many different epithelial cells, the impairment of which causes dysregulation of epithelial fluid secretion and thickening of the mucus. This, in turn, leads to the dysfunction of organs such as the lungs, pancreas, kidney and liver. The CFTR protein is mainly localized in the plasma membrane; however, there is a growing body of evidence that it is also present in the intracellular organelles such as the endosomes, lysosomes, phagosomes and mitochondria. Dysfunction of the CFTR protein affects not only the ion transport across the epithelial tissues, but also has an impact on the proper functioning of the intracellular compartments. The review aims to provide a summary of the present state of knowledge regarding CFTR localization and function in intracellular compartments, the physiological role of this localization and the consequences of protein dysfunction at cellular, epithelial and organ levels. An in-depth understanding of intracellular processes involved in CFTR impairment may reveal novel opportunities in pharmacological agents of cystic fibrosis.

## 1. Introduction

Continuous mass and energy transfer between the cells and their environment is necessary for a sustained life. Each cell is surrounded by plasma membranes delimiting the cell from its environment. Lipid bilayers are impermeable to water soluble substances, including ions; therefore, a complex system of membrane transport proteins including ion channels, transporters and pumps evolved to enable the fluxes into and out of the cell. Ion channels regulate several cellular, organellar and physiological processes such as maintaining the membrane potential, muscle contraction, neuronal signaling, calcium homeostasis, regulation of the osmolyte secretion, cell volume and pH regulation, among others. However, the activity of ion channels is restricted not only to the plasma membrane, of which the surface area represents 2–5% of the total area of cell membranes, but it is also indispensable in the functioning of many intracellular organelles [1].

Intracellular ion channels play a vital role in cellular and organ physiology by maintaining and regulating the ionic homeostasis of intracellular organelles. They were discovered and widely described in the endoplasmic reticulum (ryanodine receptors, RyRs; inositol triphosphate receptor, IP3R) [2], lysosomes (Na^+^ and K^+^ selective channels; lysosomal Ca^2+^ channels; mucopilin subfamily of transient receptor potential channels, TRPMLs) [3] and nucleus (nuclear pore complexes, NPCs; Ca^2+^-ATP-ase) [4]. There is also a wide range of ion channels present in the mitochondria, both in the outer membrane (voltage-dependent anion channel, VDAC; mitochondrial apoptosis-induced channel, MAC; translocase of the outer membrane, TOM; peripheral mitochondrial benzodiazepine receptors, PBR) [5] as well as in the inner mitochondrial membrane (uncoupling proteins, UCPs; mitochondrial Ca^2+^ uniporter, MCU; ryanodine receptors, RyRs; permeability transition pore, PTP [5]; ATP-regulated potassium channel, mitoK_ATP_ [6]; large conductance Ca^2+^-activated potassium channel, BK_Ca_ [7]; voltage-gated potassium channel, mitoKv1.3 [8]; anion channels such as the inner membrane anion channel—IMAC—or the mitochondrial chloride channel) [9].

Chloride channels are a structurally and functionally highly diverse group of anion channels that are likely present in every living cell. Plasma membrane chloride channels, acting solely or in cooperation with other ion-transporting proteins, regulate the ionic homeostasis, cell volume, intracellular and extracellular pH, transepithelial salt movement and stabilization of the cell membrane potential. Chloride channels can be classified as members of the CLC subfamily (involved in many physiological processes such as the regulation of the membrane resting potential of skeletal muscles (ClC-1), water absorption in the intestinal epithelia (ClC-2), transepithelial transport in the kidney (various ClCs) and regulation of ion homeostasis of intracellular vesicles (ClC-3,4,5,6,7)), cystic fibrosis transmembrane conductance regulator (CFTR), calcium-activated chloride channels (CaCC), maxi-anion channels known as a prostaglandin transporter, volume-regulated channel (VRAC), ligand-gated anion channels such as GABA and the glycine receptor [10,11,12,13]. The most recently discovered, classified separately from other Cl^−^ channels, are chloride intracellular channels (CLICs); they are encoded by six different genes (*Clic1–6*) and six homologues were found in mammals (CLIC1–6). The unique feature of CLIC proteins is their ability to exist as both monomeric soluble proteins and as membrane-associated proteins. This dimorphic existence suggests that CLIC proteins play many differential physiological roles. The soluble CLIC forms were shown to have enzymatic activity, which might be important in terms of protecting the cells against oxidation [13]. How these proteins form functional ion channels is not completely understood, but they are known to be present in the intracellular membranes [9,14,15,16,17]. CLICs are localized in various organelles: the endoplasmic reticulum, nucleus, endoscopes, lysosomes, secretory vesicles, mitochondria, peroxisomes, the Golgi apparatus and phagosomes [18,19]. It is noteworthy that even the name “CLIC” implies that the channels conduct chloride ions, the electrophysiological characterization of the CLIC1 protein shows poor anion selectivity, and even the almost equal permeability by K^+^ and Cl^−^ of CLIC4 and CLIC5 proteins [20,21].

Chloride channels are associated with various pathological disorders. Dysfunction of chloride channels leads to a number of pathophysiologic conditions and diseases, such as heart failure, cardiac arrhythmogenesis and myocardial hypertrophy [22,23]. CLIC dysfunction may lead to many pathological conditions such as cancer initiation, pulmonary hypertension, hearing impairment, Alzheimer’s disease and cardiac dysfunction [24]. The best known manifestation of chloride channel dysfunction, namely CFTR, is cystic fibrosis; however, it is also known that CFTR plays a role in cardioprotection against ischemia/reperfusion injury [25] and the development of CF-related diabetes [26].

CFTR is mainly known for its function in the plasma membrane and the impact on the secretory activity of the cells, yet it is also thought to be present in the intracellular organelles, a common phenomenon for all classes of ion channels. The role of CFTR in the intracellular compartments was summarized in 1999 by Bradbury [27]; however, there have been many new findings over the last 20 years. In this review, an updated characterization of CFTR is presented, with the focus on intracellular localization and function.

## 2. Structure and Function of CFTR in Cystic Fibrosis

Cystic Fibrosis Transmembrane Conductance Regulator (CFTR, also known as ABCC7) belongs to the ATP-binding cassette (ABC) transporters, the superfamily of integral membrane proteins responsible for transporting a wide variety of substrates in an energy-dependent manner [28]. CFTR is the only known member of the ABC superfamily which functions as an ion channel, transporting anions down their transmembrane electrochemical potential gradients [29,30]. It is one of the most investigated ABC transporters because the mutations in the *Cftr* gene lead to the most common lethal genetic disorder among Caucasians, namely cystic fibrosis [31].

The CFTR gene, located on chromosome 7 (7q31), encodes 1480 amino acids long channel proteins with a semi-symmetrical structure [32,33]. The CFTR channel shows the characteristic topology of the ABC protein superfamily: it is composed of two transmembrane domains (TMDs, also called MSDs) comprising six transmembrane helices connected by cytosolic and extracellular loops, as well as two cytosolic nucleotide binding domains (NBDs) with ATP-binding sites at their interface and—uniquely among the ABC protein superfamily—the regulatory domain (R-domain) between NBD1 and TMD2 that contains 18 potential phosphorylation sites [34]. R-domain phosphorylation by cAMP-dependent protein kinase A (PKA) and/or protein kinase C (PKC) is crucial to the channel activity [35]. The phosphorylation of R-domain changes the affinity of CFTRs for ATP and consequently allows the channel gating [36,37,38].

CFTR functions mainly as an anion-selective channel (permeability ratio P_Na_/P_Cl_~0.03) with a small linear conductance (6-10pS) [39]. The activated channel is permeable for various halide ions (the permeability sequence is Br^−^ > Cl^−^ > I^−^ > F^−^), but studies have shown that Cl^−^ has the highest conductance [40]. In addition, CFTR is also permeable to polyatomic anions such as NO_3_^−^, HCO_3_^−^, SCN^−^ and glutathione [41,42,43]. The capacity of CFTR for HCO_3_^−^ secretion drives the fluid transport across epithelial surfaces (i.e., in the gastrointestinal tract [44]), allows for the proper mucin expansion [45] and influences the local pH, which is critical to the proper functioning of many organs [46]. Bicarbonate, glutathione and thiocyanate anions also play an important role in killing bacteria. Glutathione is a major oxidant and its transport via CFTR may influence the redox state of the luminal and cellular compartments. Its disturbances may lead to oxidative stress, apoptosis, NF-κB activation and inflammation of the airway. In turn a thiocyanate anion is a precursor for a hypothiocyanate anion, which exhibits antimicrobial properties. Accordingly, the loss of CFTR function causes organ pathophysiologies on different backgrounds [43,47,48,49,50]. It is noteworthy that CFTR not only acts as an anion channel, but also as a regulator of other ion channels such as the epithelial sodium channel (ENaC) [51], calcium-activated chloride channels (CaCCs) [52] and exchangers from the SLC26 family [53].

Mutations in the gene encoding the CFTR protein leads to cystic fibrosis (CF), which affects at least 100,000 people worldwide. Initially, the disease was attributed primarily to Caucasian populations, but there is an increasing number of reports showing that cystic fibrosis is not ethnically linked [54]. There are more than 2100 known *CFTR* gene variants listed in the CFTR1 database (http://www.genet.sickkids.on.ca, accessed on 26 September 2021); however, many of them may not elicit a defect in CFTR mRNA, protein or cause clinical symptoms [55]. Recent data (31 July 2020) from Clinical and Functional Translation of CFTR (CFTR2 Database, http://www.cftr2.org, accessed on 26 September 2021) annotate 442 variants, of which 360 have confirmed disease liability and 48 variants with varying demonstrated clinical consequences. The most common CF-causing mutation is the deletion of phenylalanine at position 508 (F508del) affecting approximately 70–90% of CF patients [55,56]. The mutations were grouped according to their effect on protein synthesis, function or stability. Traditional classification distinguishes six classes of mutations: Class I—defective protein production (reduced or absent CFTR expression); Class II—defects in CFTR processing (premature protein degradation, impaired protein biogenesis); Class III—impaired CFTR channel regulation (abnormal channel gating and reduced open probability); Class IV—reduced channel conductance (obstructed conduction pore); Class V—reduced number of channels present at the membrane (affected pre-mRNA splicing, defective CFTR production); and Class VI—decreased stability of CFTR at the plasma membrane (increased endocytosis or decreased recycling to plasma membrane). Mutations from classes I–III are considered more severe forms of CF than those from classes IV–VI due to the lack of residual CFTR function [57]. The new classification of *Cftr* mutations on the basis of therapeutic strategies was suggested by Kris De Boeck and Margarida Amaral, who proposed to divide Class I into two classes: Class I including stop codon mutations and a new Class VII comprising so called “non-rescuable” mutations (such as large deletions) that cannot be pharmacologically corrected as there is no mRNA transcription [58]. Since the proposed classification does not take into account the severity of the mutations, Ribeiro et al. proposed to divide the traditional Class I into two subtypes: Class IA (no mRNA transcription) and Class IB (no protein synthesis) [59].

Cystic fibrosis is mainly related to secretory epithelia, such as sweat glands, airways, the pancreas, the gastrointestinal tract and vas deferens [60]; however, the CFTR channels were also found in non-epithelial cells such as blood, the heart and the brain as well [61,62,63,64,65]. The Human Protein Atlas provides useful information on CFTR localization in organs, tissues and cells. It indicates that CFTR is predominantly present in the pancreas, but also in the liver, salivary glands, the gastrointestinal tract, the lungs and the male and female reproductive system. The presence of CFTR in these tissues and organs was confirmed in epithelial and endocrine cells. Moreover, it was detected on the RNA level in immune cells, such as granulocytes and monocytes [66]. It was observed that CFTR dysfunction not only affects the secretory functions of organs, but also regulates many intracellular processes such as chemokine regulation [67] and glucose metabolism [68], among others. Moreover, the localization and role of CFTR may be linked not only to the plasma membrane, but also to subcellular locations and interference with other intracellular channels [69].

## 3. Intracellular Trafficking of CFTR

CFTR undergoes complex co- and post-translational processing. CFTR folding starts early during translation and requires several folding machineries. The process occurs in a modular manner (domain by domain) where the transmembrane segments are inserted into the endoplasmic reticulum (ER) membrane simultaneously. The first step of CFTR folding is represented by the positioning of the MSD1 domain into the ER membrane [70] followed by the synthesis of the NBD1 domain. During the R-domain synthesis, NBD1 is bound transiently by chaperons (Hdj2 and Hsc70) that promote its folding and stabilization [71]. Synthesis and integration into ER of the MSD2 domain leads to the release of most chaperones and MSD1–MSD2 complex formation. The final step is represented by NBD2 domain synthesis followed by post-translational maturation of CFTR conformation [72,73]. CFTR proteins that are folded and assembled properly are transported to the Golgi apparatus, where they undergo further maturation and glycosylation. The mature channels are packed into secretory vesicles and transported to the plasma membrane [74].

Since the process of folding and domain assembly is error prone it is monitored by a quality control system, ensuring that only correctly folded CFTR proteins reach their final destination. There are many cellular checkpoints during CFTR biogenesis that seem to be a target for pharmaceutical intervention, which were recently reviewed by Estabrooks and Brodsky [74]. Misfolded proteins are degraded mainly by the ERAD system (endoplasmic-reticulum-associated protein degradation), the principal quality-control mechanism responsible for the recognition and clearance of misfolded proteins trapped in the ER. Incorrectly folded proteins are recognized by ER chaperones, ubiquitinated and delivered to the proteasome for degradation (also called ubiquitin proteasome system, UPS) [75]. There are also unconventional pathways of CFTR secretion, namely ESCRT (endosomal sorting complex required for transport), the route that bypasses the Golgi apparatus [76]. This route belongs to type IV of unconventional protein secretion (UPS) [77]. It is mediated by Golgi reassembly stacking proteins (GRASPs) which interact with both wtCFTR and F508del CFTR, in conditions of ER stress or ER-to-Golgi block [78]. It was also suggested that a key role in the process is played by autophagosome formation, multivesicular body (MVB) development and RAB8A GTPase-dependent recycling vesicle transport to the plasma membrane [76].

CFTR is present not only in the plasma membrane, but also in various cellular compartments. The proposed mechanism for the presence of CFTR in the intracellular membranes is membrane vesicle trafficking. Basically, the fragment of the membrane containing CFTR protein is internalized and transported within the cell [27]. It was estimated that as much as 50% of the plasma membrane CFTR is internalized within a few minutes by endocytosis through clathrin-coated vesicles [79,80].

## 4. CFTR Intracellular Vesicles: Focus on Endosomes, Golgi, Lysosomes and pH

The first reports showing the correlation between chloride anion transport and the acidification of the intracellular vesicles were made in the late 1970s and 1980s. Barasch et al. demonstrated that in secretory granules of parafollicular cells, the transport of chloride anion compensates for the charge in the vesicles, which is necessary for H^+^ translocating ATP-ase [81]. Later it was discovered that cystic fibrosis cells also show the defective acidification of such organelles as the Golgi network, prelysosomes and endosomes, due to reduced Cl^−^ conductance [82]. Bradbury proposed that CFTR plays an important role in the intracellular vesicles as it regulates exocytosis and endocytosis, intravesicular trafficking and pH [27]. The role of CFTR in the intracellular vesicles is broadly explained below and summarized in Table 1.

A great deal of emphasis was placed on endosomal acidification in terms of proper kidney functioning. Endosomes of kidney epithelial cells play a crucial role in the albumin and low-molecular weight plasma protein filter system. It was shown that the localization of CFTR, which is highly expressed in kidney epithelium, is compatible with the localization of endosomes. It was also found that, in endosomes, CFTR has a similar localization to ClC-5 and V-ATPase and that CFTR-deficient mice display defective endocytosis [83,84]. The role of the chloride channels, CLCs and CFTR, concerning this physiological activity was reported [85]. Briefly, vesicular acidification drops from pH 6.0 in the early endosome, to pH 5.5 in the late endosome, to pH < 5.0 in the lysosome [86]. In parallel, the concentration of Cl^−^ increases from 20–40 mM, to 60 mM, to more than 80 mM. It is suggested that ClC-5 acting as a Cl^−^H^+^ exchanger supports acidification and CFTR participates in Cl^−^ transport into the vesicle lumen. This provides the electrical shunt and sustains proper acidification driven by V-ATPase (vacuolar ATPase) [85].

Proper acidification is also required in lysosome enzymes activated at low pH [87]. The enzymes cleave the macromolecules into smaller particles, a process that is inevitable in cell function, for example in retinal pigmented epithelial (RPE) cells. It was reported that CFTR activation decreases pH level in lysosomes from alkaline to acidic [88]. The degradation system in RPE cells is required for a reduction in photoreceptor outer segments. It is essential to keep retina transparent, and all the subretinal deposits that are not lysed in lysosomes prevent transparency. This in turn may lead to age-related macular degeneration (AMD). It was observed that CFTR activation increases degradation due to the restoration of pH in lysosomes [89].

**Figure 1:**
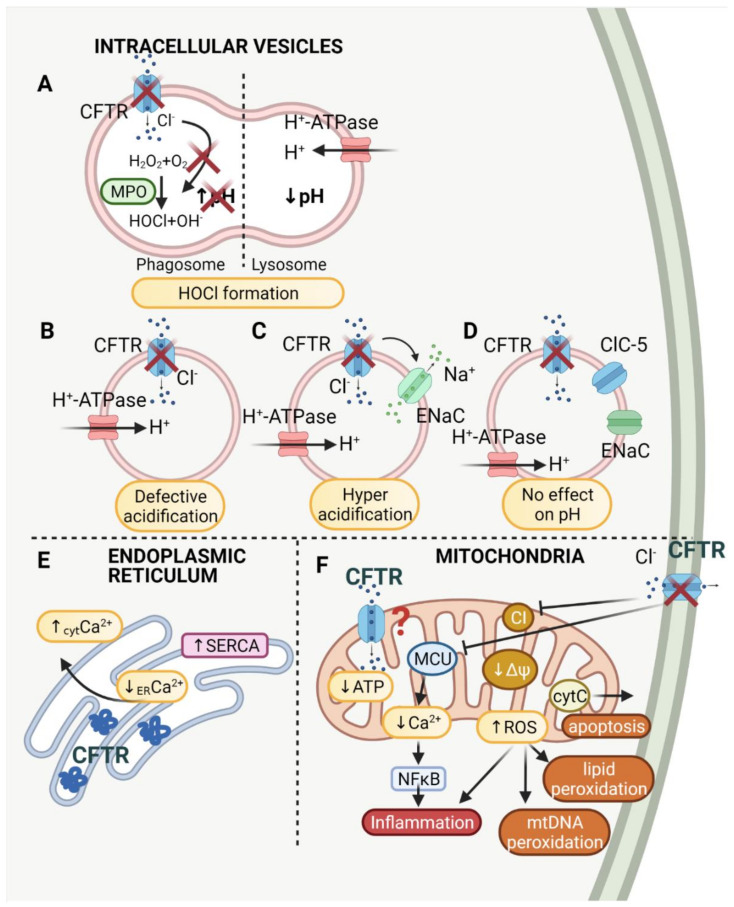
Summary of CFTR role in the intracellular organelles. (**A**–**D**) show the effect of CFTR impairment on intracellular vesicles. In phagosomes (**A**) of CFTR-depleted cells HOCl formation from Cl^−^, O_2_ and H_2_O_2_ by myeloperoxidase (MPO) is impaired. In healthy cells the process results in a phagosomal pH increase which is compensated by low pH of lysosomes fusing with phagosomes to form phagolysosomes. Changes in pH of other intracellular vesicles (lysosomes, Golgi) varies. There are three different pathways indicated. (**B**) CFTR malfunction may lead to defective acidification due to a deficiency in the counter anion transport of Cl^−^, required for proper H^+^−ATPase function and low pH maintenance. (**C**) Another mechanism of CFTR malfunction involves the lack of CFTR inhibitory effect on endothelial sodium channel (ENaC), which promotes outward sodium cation transport and results in hyperacidification. (**D**) No effect of CFTR on vesicle pH indicates other channels involved in proper acidification such as ClC-5. (**E**) In cystic fibrosis there is increased concentration of misfolded CFTR protein in the endoplasmic reticulum, which causes increased SERCA activity and the release of Ca^2+^ from ER to cytoplasm. (**F**) The presence of CFTR in mitochondria is poorly documented. The effect of plasma membrane CFTR depletion on mitochondria is associated with: complex I (CI) of electron transport chain inhibition, lower ATP production, a decrease in mitochondrial membrane potential (Δψ), MCU inhibition and cytochrome c release. The consequence of MCU inhibition is Ca^2+^ release and NFκB activation, which leads to inflammation. Besides Ca^2+^, ROS generated by mitochondria of CFTR-depleted cells also mediate inflammation as well as mitochondrial DNA and lipid peroxidation. Created with BioRender.com.

Vacuolar pH and Δψ is mainly generated by vacuolar proton pumps (ATP-ases). It was suggested that the membrane potential generated by proton pumps inhibits further proton transport and therefore prevents vesicular acidification. The inward chloride anion transport is supposed to dissipate the gradient and allow acidification (Figure 1B). Direct measurements of endosomal Cl^−^ show that endosomal acidification corresponds to inward chloride anion transport and an increase in endosomal volume [90]. It was proven that chloride channels, such as CFTR, are present in endosomes in Chinese hamster ovary (CHO) cells; however, the authors question the role of the counter ion conductance as the primary factor limiting acidification of the vesicles [91]. Similarly, it was shown that Golgi acidification was increased in CFTR-deficient epithelial cells, but the difference compared to healthy cells was slight and presumed to be physiologically unessential [92].

The CFTR-dependent acidification was questioned by many different laboratories. The hypothesis was not confirmed in endosomes of enteroendocrine cells [93], lysosomes of the pancreatic adenocarcinoma cells [94,95], trans-Golgi and endosomal compartments of fibroblasts [96], the trans-Golgi network of kidney cells [97] and the Golgi of HeLa and airway epithelial cells [98]. All the above-mentioned experimental data suggested that CFTR does not play a role in endosomal and Golgi pH regulation. Neither CFTR channel activation nor its inhibition influenced the pH in endosomes and phagosomes in mouse alveolar macrophages and human respiratory epithelia [99].

Contradictory results were shown for endosomes and Golgi from bronchial and lung epithelial cells. It was demonstrated that CFTR is involved in the alkalization of the vesicles. By means of the use of the CFTR channel activator, forskolin, it was established that CFTR is involved in the alkalization of endosomes from fibroblasts and epithelial cells [100]. Furthermore, CFTR dysfunction also led to the hyperacidification of the trans-Golgi network in bronchial epithelial cells [101]. The pH discrepancies between studies may be explained by CFTR–sodium channel interactions. It was demonstrated that proper acidification in the endosomes of lung epithelial cells depends on sodium transport. Organellar hyperacidification results from the loss of the inhibitory effect of CFTR on sodium transport [102]. It was proposed that CFTR inhibits the epithelial sodium channel (ENaC) in endosomes, which results in the proper acidification of the vesicles. CFTR deficiency causes a sodium efflux from the vesicle as ENaC is no longer inhibited. The sodium efflux results in potential dissipation and increased ATP-ase activity which pumps protons into the vesicle lumen and allows for higher acidification of the endosome [103] (Figure 1C).

The localization of CFTR in endosomes was also confirmed in the intestine cells using immunohistochemistry [104] and immunogold labeling [105]. The proposed localization of CFTR was associated with CFTR trafficking within the cell as cAMP induced CFTR vesicle insertion in the intestine. Further studies on intestine cells showed that intracellular pH was also decreased after the inhibition of CFTR [106].

Different results concerning pH in the intracellular vesicles still require further investigation in order to elucidate the mechanism. The results may be associated with different models, cell types and methods employed. The function of the CFTR protein in the intracellular compartments is summarized in Table 1.

**Table 1:** The role of CFTR in the intracellular vesicles shown in the context of the source material.

Function	Organelle	Material	Reference
**CFTR sustains lower pH**	Golgi network Lysosomes Endosomes Phagosomes Lysosomes	CF nasal polyps HBE, CFT1, HeLa, JME T84 (human colon carcinoma) cells; Swiss 3T3 fibroblasts Primary monocyte-derived macrophages (MDMs); human isolated neutrophils; isolated murine alveolar macrophages; J774 (murine macrophage-like cell line); raw 264.7 (murine macrophage cells) RPE (retinal pigmented epithelial cells); ARPE−19 cells; murine bronchial epithelial cells; pancreatic adenocarcinoma cell line (CFPAC−1)	[82] [92] [100] [107,108,109] [110] [88,89,94,111]
**CFTR sustains higher pH**	Golgi network Endosomes	IB3−1 (human bronchial epithelial cell line derived from CF patient); C38, S9 (control cell lines); CFT−1 (cell line derived from tracheal epithelium of a CF patient)	[101,102,103]
**No effect of CFTR on pH**	Golgi network Endosomes Phagosomes Lysosomes	HeLa; airway epithelial cells (CFT1, CFT1−CFTR), Madin–Darby canine kidney (MDCK) cells, Swiss 3T3 fibroblasts, Calu−3 cells, SK−MES−1 cells; mouse alveolar macrophages, baby hamster kidney (BHK) cells; pancreatic adenocarcinoma cell lines (CFPAC−1); intestinal endocrine cells (L cells); monocytes derived from PBMC; murine alveolar macrophages; J774A.1 cell line; human and murine isolated epithelial cells; primary cultures of human airway epithelial cells; murine macrophages: RAW264.7; J774	[93,95,96,97,98,99,112,113,114,115]
**ROS production**	Phagosomes	Isolated murine alveolar macrophages; J774 (murine macrophage-like cell line)	[109]
**Cl^−^ transport into vesicle lumen** **HOCl formation**	Phagosomes	Human PMN (polymorphonuclear) neutrophils; human peripheral blood neutrophils; murine peripheral blood neutrophils	[116,117,118,119,120,121]
**Ca^2+^ homeostasis**	Endoplasmic reticulum	CFBE, HBE (human bronchial epithelial cells)	[122,123]

## 5. CFTR in Phagolysosomes: pH Implications for Bacteria Killing

Cystic fibrosis is a severe disease associated with serious lung infections. Bacteria killing and the clearance thereof by means phagocytes, such as macrophages, are essential to prevent these infections. Phagocytosis involves the fusion of the phagosome and lysosome, which form the phagolysosome. It has been shown that neutrophils of CF patients’ phagocytes have lower phagocytic capacity, which was not associated with ATP or calcium ion level [124]. It was also observed in the CF tracheal epithelial cell line CFT-1 that *Staphylococcus aureus* escapes from the formed phagolysosomes [125] and that *Burkholderiacenocepacia* survives in the phagolysosomes of CF macrophages [126]. Interesting observations were made of *Staphylococcus aureus*, which was properly internalized by CFTR-deficient lung macrophages but not killed [127], and also for *Pseudomonas aeruginosa* in PBMC interactions, which showed that the absence of CFTR did not affect phagocytic activity, but resulted in the reduction in bactericidal activity [128]. The localization of CFTR in phagosomes was confirmed in PBMC as it was shown that F508del–CFTR mutants failed to target the structures [129]. Decreased phagocytosis was observed along with increased apoptosis; however, CFTR modulators (Ivafactor) modestly improved bacteria killing in macrophages [130].

It was proposed that infection susceptibility in CF is correlated with lysosomal pH [111], and confirmed that in alveolar macrophages CFTR helps to maintain lysosomes at low pH, a phenomenon required for effective inhibition of bacteria growth [131]. It was demonstrated that CFTR was localized in perinuclear regions, the Golgi apparatus, as well as phagolysosomes of the J774 macrophage cell line. Additionally, phagosomes of CFTR-/- alveolar macrophages were over 1 pH unit more alkaline than controls [132]. The proposed mechanism suggests that the lysosomes that fuse with phagosomes containing internalized bacteria, responsible for maintaining low pH and CFTR-/- cells, show defective lysosomal acidification (Figure 1B). Later it was demonstrated that the loss of CFTR function causes defective acidification in endosomal, lysosomal and phagosomal compartments of alveolar macrophages, resulting in a decreased phagocytic response [133]. The alkalization of lysosomes was also confirmed in CFTR-deficient macrophages [109]. CFTR localization in lysosomes of macrophages was demonstrated. Recent studies also support the concept of defective acidification in CFTR-deficient cell phagosomes, as CFTR inhibition was shown to increase the vesicle pH in human macrophages 07] and neutrophils [108].

The hypothesis of defective phagosomal acidification in CFTR-depleted cells was also much disputed. Recently it was shown that there is no defect in the acidification of cystic fibrosis PBMC phagosomes [112]. These results are in accordance with previous observations indicating that the CFTR inhibitor did not change the pH of phagosomes in macrophages [113] and that the lysosomal pH of CF and non-CF airway epithelial cells (of different origin) seemed unchanged [114]. The complex mechanism of ceramide accumulation due to changes in lysosomal pH and consequent CF lung disease was reviewed elsewhere [134]. The discrepancies between the results from different lab groups may be linked to different CFTR distribution between cells. It was demonstrated that CFTR is expressed in monocytes but not in neutrophils, and that phagocytosis and bacteria killing of *Pseudomonas aeruginosa* is impaired in CF monocytes isolated from peripheral blood [135]. It was also suggested that, in addition to chloride anions, cations can support lysosomal acidification [115]. Other mechanisms are needed to explain the phenomena such as enhanced bacterial cellular survival, which was observed alongside normal acidification in phagosomes of macrophages [110]. Macrophage dysfunction in the context of cystic fibrosis is a much disputed topic, and some other factors that may be involved such as different types of mutations and different types of macrophage models should be taken into consideration [136]. Another aspect of investigations that needs to be considered is that commonly used activators and inhibitors of CFTR, namely CFTR_inh_-172 and GlyH-101, may exert non-specific effects, which are independent of the CFTR function [137].

## 6. CFTR in Phagolysosomes: HOCl Production

Phagocytic activity is widely correlated not only with proper phagolysosomal pH, but also with hypochlorous acid (HOCl) production. The mechanism is thoroughly described by Roos and Winterbourn [138]. Briefly, HOCl is formed in phagosomes by myeloperoxidase (MPO) from H_2_O_2_ and Cl^−^ with OH^−^ byproduct, which may lead to alkalization [139]. However, it was also proposed that the fusion of granules with phagosomes lowers pH and increases vesicle volume. These two distinct mechanisms of pH regulation in phagosomes may contribute to discrepancies in pH measurements (Figure 1A).

The expression of CFTR in phagolysosomes was confirmed in neutrophils, as argued in the later work of Van de Weert-van Leeuwen, which revealed no expression of CFTR in human neutrophils. However, the ambiguous nature of samples isolated from human patients must be considered. The expression of CFTR in phagolysosomes was linked to proper intraphagolysosomal HOCl production [116]. The killing rate of *Pseudomonas aeruginosa* in neutrophils was lower in CF regardless of the chloride environment [140] and the role of CFTR in proper Cl^−^ transport into the phagosomes was shown to be crucial [120]. It was also shown that CFTR plays a crucial role in the chloride anion transport needed for HOCl formation [121]. Moreover, the targeting of CFTR to subcellular sites in F508del neutrophils is impaired, which diminishes HOCl production [118]. Impaired phagosomal HOCl production in turn reduces microbial killing and leads to CF lung infections [117], a phenomenon confirmed by recent studies on CF neutrophils [119].

## 7. CFTR and Mitochondria

Mitochondria are well-known organelles that produce ATP, an energy-carrying compound essential to living cells. However, nowadays it is well established that mitochondria also play a critical role as signaling organelles through control of the cellular calcium level, production of free radical species and an active role in apoptotic and necrotic cell death [141]. Calcium uptake by mitochondria regulates the calcium signals within the cells and therefore exerts many cellular signaling pathways [142]. Reactive oxygen species, another powerful signaling molecule, are also produced in mitochondria, in both the matrix and electron transport chain, namely in complex I and III of the respiratory chain [143]. ROS and redistribution of oxygen, in turn, implicate the role of mitochondria in oxygen sensing via the HIF (hypoxia inducible factor) pathway [144]. Last but not least, modulation of calcium signaling and ROS by mitochondria leads to apoptosis, a programmed cell death [145]. The regulatory role of mitochondria is strongly related to the presence of mitochondrial ion channels, especially potassium channels. Their role in many cytoprotective events (e.g., against ischemia/reperfusion injury) and in cell death is widely documented [146]. Besides the potassium channels, there are several anion channels presenting the mitochondrial membranes: VDAC (voltage dependent anion channel in the outer mitochondrial membrane), IMAC (inner membrane anion channel), mitochondrial CLIC proteins, mPTP (mitochondrial permeability transition pore), maxi ClC (maxi chloride channel) and UCPs (uncoupling proteins) [9,147].

The relationship between cystic fibrosis and mitochondria was first observed in 1982 by Shapiro et al., who suggested an association between CF and mitochondrial NADH dehydrogenase [148]. However, it was later discovered that CFTR, which is responsible for CF, is a chloride channel. Subsequent studies showed that the expression of the mitochondrial gene encoding ND4 subunit of the mitochondrial complex I is downregulated in cystic fibrosis [149], which was confirmed by the measurement of mitochondrial complex I activity [150]. The reduction in complex I activity has many effects within the cell, for example an increased ROS level, reduced ATP synthesis and apoptosis (Figure 1F).

Indeed, CF cells have markedly increased ROS levels. The dysregulation in redox state is revealed by oxidative stress, as well as levels of glutathione (GSH), peroxiredoxin and SODS [19]. Defective CFTR leads to mitochondrial ROS production together with NOX. CF cells were shown to have an increased expression level and activity of NOX, increased ROS production, as well as a decreased glutathione level, both intracellular and extracellular [151]. The effect of CFTR mutation on mitochondria is manifested not only by aberrant ROS production and related lipid membrane peroxidation, but also by changes in oxygen consumption, reduced mitochondrial membrane potential (ΔΨ), ADP/ATP exchange and complex I and IV activity [152]. Additionally, it impairs mitochondrial Ca^2+^ uptake and induces fragmentation of the mitochondrial network. Together with the high level of glucose in ASL (airway surface liquid), it indicates the prominent role of mitochondria in cystic fibrosis [153]. Its role in lowering both ROS and ASL glucose levels was proven recently using different compounds reducing mitochondrial functions [154]. Dysfunction of mitochondria in CF cells and impaired redox state lead to certain characteristic symptoms of CF such as inflammation and autophagy [155]. The use of CFTR correctors is associated with the rescue of some mitochondrial functions [156]. It has recently been shown that CFTR impairment is strongly associated with the modification of mitochondrial morphology in bronchial epithelial cells [157].

Interestingly, not only lung and bronchial epithelial cells have mitochondrial dysfunction related to CFTR abnormalities. In the cardiac hypertrophy induced by high fructose, CFTR expression decreased and CFTR silencing resulted in mitochondrial oxidative stress [158], whereas in intestinal epithelial cells, CFTR deletion resulted in lipid homeostasis disruption and mitochondrial changes such as reduced cytochrome c level, lowered expression of OXPHOS complexes and a high ADP/ATP ratio [159,160]. In smooth muscle cells, CFTR deletion also caused mitochondrial dysfunction, which led to apoptosis [161]. Additionally, in the neuronal system, namely brain tissue, CFTR activation protected the cells from apoptosis induced by ischemia/reperfusion events and oxidative stress, whereas CFTR loss led to mitochondrial oxidative stress [162].

Along with changes in the redox state of CF cells, there is a prominent change in Ca^2+^ signaling. Depolarization of mitochondria in F508del cells led to a dysfunction of MCU (mitochondrial calcium uniporter) and reduction in Ca^2+^ uptake by mitochondria [163]. The mitochondrial structure in CF cells was disrupted and mitochondrial Ca^2+^ seems to control the inflammatory response. It was discovered that MCU is a signal effector that drives the NLRP-3 activation via Ca^2+^, inflammasome formation and cytokines release (IL1β, IL-18) [164,165], thus indicating the role of CF cell mitochondria in the development of inflammation.

The crosslink between mitochondria, inflammation and immunity is well established [166]. Mitochondria are involved in many aspects of host responses such as extracellular and intracellular bacterial infections, virus replication and dissemination or the development of innate immunity [167]. Therefore, mitochondria are strongly involved in the inflammatory response in CF patients. Mitochondrial stress in CF includes: (i) reduced mitophagy and accumulation of dysfunctional mitochondria; (ii) impaired UPR (unfolded protein response) leading to upregulation of immune-response genes; (iii) mitochondria-driven apoptosis and mtDAMP accumulation, which is strongly related to changes in ROS level and Ca^2+^ uptake by mitochondria. All the mitochondrial changes lead to hyper-inflammation, cytokine production and inflammasome upregulation [168] (Figure 1F).

In the view of such diverse mitochondrial function dysregulation in cystic fibrosis, the role of CFTR should not be neglected. Furthermore, through the use of the Western blot technique, it was recently shown that CFTR is also located in the mitochondrial membrane [162]. However, the localization of CFTR in mitochondria requires further investigation using electro-physiological techniques, such as the patch–clamp method of the mitochondrial membrane.

## 8. CFTR and the Endoplasmic Reticulum

CFTR mutations lead to abnormal Ca^2+^ homeostasis, which is correlated not only with mitochondrial dysfunction, but also, and mainly, with abnormal endoplasmic reticulum function. Retention of misfolded CFTR protein in the endoplasmic reticulum leads to ER condensation, IP3R (inositol triphosphate receptor) clustering in ER and Ca^2+^ release from ER stores. At the plasma membrane level, the absence of CFTR protein leads to TRPC6 (transient receptor potential cation channel) hyperactivity, which enhances the cytoplasmic Ca^2+^ level [169]. Experimental data from CF cells show increased SOCE (store operated Ca^2+^ entry) and release of Ca^2+^ from ER (Figure 1E). In parallel, PMCA (plasma membrane Ca^2+^ ATPase) activity is decreased, whereas SERCA (sarcoplasmic/reticulum Ca^2+^ ATPase) activity is increased [122]. It was also found that ER related Ca^2+^ homeostasis is modulated by the Ca^2+^ binding protein Calumenin (CALU), which indicates the potential therapeutic use of Calumenin in CF [123].

CFTR accumulation in ER was observed in healthy cells exposed to toxic factors, such as cigarette smoke. It was discovered that, upon cigarette smoke exposition, plasma membrane CFTR undergoes retrograde trafficking to the endoplasmic reticulum [170]. This impairs CFTR function and leads to the dehydration of the lungs and consequently airway diseases, such as chronic obstructive pulmonary disease (COPD).

## 9. Intracellular Crosstalk and Interdependencies

Most studies on the role of the CFTR protein in intracellular compartments are focused on each organelle separately. However, the additional in-depth understanding of the signaling pathways between the intracellular organelles is critical to comprehension of cystic fibrosis.

For mitochondria, calcium homeostasis in the cell is essential to inflammatory processes [164]. Perturbations of calcium delivery may lead to autophagy and mitophagy. What is more, in these processes the communications between endoplasmic reticulum and mitochondria were shown to play a very important role. It was observed that bacterial infection of CF cells caused VAPB (ER vesicle-associated membrane protein-associated protein B) and PTPIP51 (outer mitochondrial membrane protein tyrosine phosphatase interacting protein 51) tether tightening and expression and consequently impairment of autophagy. Interestingly, MCU inhibition abrogates this effect [165].

Other signaling molecules, such as ROS, are frequently described in the context of CF. However, the effect of ROS on different intracellular organelles in CF progress needs further investigation. It is well described that ER channels and receptors, IP3Rs and RyR, are involved in calcium release, which in turn can activate ROS, releasing mitochondrial pathways. Less obviously, ROS is a potent signaling molecule that can regulate the function of other intracellular ion channels, such as RyR [171]. The role of lysosomes in redox balance also cannot be neglected. It was proposed that lysosomes take part in Zn^2+^, Fe^2+^ and Cu^2+^ turnover within the cell, which in turn leads to lowering the metal-dependent ROS production.

The proposed interactions show only the directions in which CF mechanisms are not clearly explained, although an understanding of the processes is needed for proper development of CF therapies.

## 10. Therapies

Cystic fibrosis affects all wet surfaces of the body; however, the most serious complications are problems with the respiratory tract. The lack of CFTR function in the lungs drives the accumulation of thick and viscous mucus leading to chronic inflammations, bacterial infections and consequently to progressive lung degradation. Since most CF patients die from respiratory failure, the lungs are the primary target of the therapies. The most common pathogens found in the lungs of CF patients are *Pseudomonas aureginosa* and *Staphylococcus aureus* (i.e., *P. aureginosa* was found in 70% of adult CF patients’ lungs) [172,173]. Currently, conventional treatment includes prophylaxis, delaying chronic infections, preventing the progression of exacerbations, preventing lung damage and treatment of acute exacerbations [174], which are mostly achieved though the chronic use of antibiotics [175].

Nowadays, different novel treatment strategies aiming to restore CFTR function, which are specific to CFTR mutation or class of mutation, are being developed. The potential therapeutics are identified by high-throughput screening drug discovery programs and tested on various cell models replicating the pathophysiological defect [176]. Today, small molecule drugs (SMDs) already approved by the FDA can bring clinical benefits to approximately 90% of CF patients carrying the most common mutations (i.e., F508del) on at least one allele gating and conductance mutations. Novel treatment strategies target the CFTR at various levels, which include increasing CFTR mRNA levels (nonsense-mediated mRNA decay (NMD) inhibitors); suppressing translation termination at premature stop codon (PTC) in CFTR mRNA (readthrough therapies); correction of CFTR folding and trafficking to apical membrane (correctors); increasing the channel function (potentiators); stimulation of CFTR expression (amplifiers); and increasing the stability/decreasing degradation rate of CFTR protein at the plasma membrane (stabilizers) [177,178]. Some of the best known SMDs are CFTR corrector VX-809 (lumacaftor) for class II mutations and CFTR potentiator VX-770 (ivacaftor) for the G551D mutation [179]. Ivacaftor (brand name Kalydeco), the first CFTR modulator approved by the FDA (2012), brought proof that CFTR protein correction can improve the health of CF patients. The ivacaftor clinical studies showed the improvement of several CF disease parameters such as forced expiratory volume in one second (FEV_1_) by 10%, a decrease in sweat chloride concentration to close to normal values and a decrease in the frequency of respiratory exacerbations. However, the ivacaftor monotherapy is limited to patients carrying gating mutations (especially G551D), which represent only about 5% of all CF-causing mutations [180]. The F508del mutation causes the production of misfolded CFTR protein rapidly undergoing ERAD. This results in few or no CFTR channels present at the plasma membrane that may have gating defects. In vitro studies showed that ivacaftor was indeed able to potentiate CFTR in F508del HBE cultures [181]; however, clinical studies of CF homozygous for F508del patients showed that the ivacaftor treatment was not associated with significant clinical benefits [182]. High-throughput screening drug discovery programs identified lumacaftor as an agent that could have an impact on misfolded protein trafficking; however, monotherapy on F508del patients led to minimal clinically relevant outcomes. The efficacy of the corrector was demonstrated in combination with ivacaftor. Clinical studies of Orkambi (combination therapy of lumacaftor and ivacaftor) showed the improvement of the FEV_1_ and reduced respiratory exacerbation of homozygous F508del patients [183]. When combined with ivacaftor, tezacaftor, an alternative CFTR corrector, also showed an improvement in CF patients (both F508del homozygotes and F508del heterozygotes), albeit displaying fewer respiratory-related adverse events [184]. Subsequent studies on the effects of three-drug combinations showed that the combination of elexacaftor (a next-generation corrector) with tezacaftor and ivacaftor resulted in significant improvement in the lung function of treated patients (it improved FEV_1_ by 14.3% and reduced sweat chloride concentration). This three-drug combination therapy (brand name Trikafta) was recently approved by the FDA (2019) for CF patients carrying at least one F508del allele, thus covering about 90% of CF patients [185]. Nowadays different CFTR modulators are being assessed in clinical trials: VX-121 (corrector, Phase 2; NCT03912233), VX-561 (potentiator, Phase 2: NCT03911713) from Vertex Pharmaceuticals, ABBV-2222 (corrector, Phase 2, NCT03969888), ABBV-3067 (potentiator, Phase 2, monotherapy and co-therapy with ABBV-2222) from AbbVie, PTI-428 (amplifier, Phase 2), PTI-801 (corrector, Phase 2) or PTI-808 (potentiator, Phase 2) from Proteostasis Therapeutics. The development of CFTR modulator therapies can bring benefits for most (approximately 90%) of CF patients. However, it is worth mentioning that such therapies may not bring benefits to all CF patients (i.e., not all patients may respond to the treatment). Additionally, the highly effective therapies are very costly (approximately $300,000 annually) and access to these agents is unequal across the world.

The CFTR correctors can have beneficial effects on the intracellular environment as well. It was documented that the VX-809 corrector molecule decreases the ROS level within the cell and improves the cell antioxidant system [151]. Additionally, it was discovered that VX-809 improved mitochondrial functions in CF cells, such as oxygen consumption rate and mitochondrial membrane potential [152]. Research on calcium homeostasis showed that VX-809 was unable to restore normal Ca^2+^ uptake by mitochondria; however, it normalized the ER Ca^2+^ release via regulation of SERCA activity (and PMCA as well) in CF epithelial cells [122]. Moreover, calcium homeostasis in CF cells was shown to be normalized by means of another corrector, miglustat, which decreased the Ca^2+^ mobilization in CF cells [186].

Another approach to CF treatment involves antioxidant therapy, as oxidative stress plays a key role in the disease, as described above. Antioxidant therapy includes inhaled GSH, sodium pyruvate, administration of anti-oxidant-rich multivitamin, NAC, curcuminoids, NO inhalation or glutamine supplementation. However, despite positive effects at laboratory level, there have been poor outcomes at the clinical trial level. This implies that a new generation of anti-inflammatory tools are needed [187].

It is still unknown whether inflammation is caused directly by the CFTR mutation or by the infection and mucus accumulation. Studies on ferret and pig models suggested that infection is not required for chronic inflammation in CF [188,189]. Bronchoalveolar liquid studies on newborn CF babies detected the inflammation in infants independently of infection [190]. It is noteworthy that the CF phenotype depends not only on CFTR mutation, but also on environmental and non-CFTR genetic factors (such as pollution, climate, economic status, microbial and particulate matter exposure) [191]. CFTR mutations resulting in mucosal immune function involve many changes in epithelial functions, such as NFκB activation, altered TLR4 trafficking, cytokine production and dysregulation of redox state [192]. This again indicates that there are many factors involved in cystic fibrosis. The future of the development of CF therapy seems to be a combination of pharmaceutical agents acting on different levels, both extracellular and intracellular. This approach needs further examination and understanding of the processes within the cell and intracellular organelles in cystic fibrosis.

## 11. Conclusions

Cystic Fibrosis is a disease affecting mainly secretory organs in humans. The disease is a genetic disorder with malfunctioning CFTR protein. The CFTR protein combines chloride channel properties and ABC transporters and its malfunction results in impaired ion and water transport across the plasma membrane. However, the effects of CFTR dysfunction are far beyond the presence of thick mucus in the secretory organs. A deficiency thereof is also observed in non-secretory cells. The hallmarks of CFTR include deficiency in the cell reactive oxygen species production, acidification changes of intracellular vesicles and dysregulation of many intracellular signaling pathways. Thus, it was observed that CFTR protein is present not only in the plasma membrane but also in other intracellular organelles such as the Golgi network, endosomes, phagosomes, lysosomes, endoplasmic reticulum and mitochondria (here some more evidence is required). The importance of understanding the intracellular pathways and intracellular localization of CFTR protein opens new targets for pharmacological intervention in cystic fibrosis patients.

## Data Availability

Not applicable.
